# The Interference of Age and Gender on Smile Characterization Analyzed on Six Parameters: A Clinical-Photographic Pilot Study

**DOI:** 10.3390/medicina59030595

**Published:** 2023-03-17

**Authors:** Joana Cunha, Gustavo Vicentis Oliveira Fernandes, Juliana Campos Hasse Fernandes, Pedro C. Lopes, Rute Rio

**Affiliations:** 1 Faculty of Dental Medicine, Universidade Católica Portuguesa, 3504-505 Viseu, Portugal; 2 Periodontics and Oral Medicine Department, University of Michigan School of Dentistry, Ann Arbor, MI 48109, USA; 3 Center for Interdisciplinary Research in Health (CIIS), Universidade Católica Portuguesa, 3504-505 Viseu, Portugal; 4 Private Practice, Ann Arbor, MI 48109, USA; juegu11@gmail.com

**Keywords:** smiling, dentistry, dental esthetics, aging, gender identity, photography

## Abstract

Background and *Objective*: This study aimed to evaluate six smile-esthetic parameters (deviation of the upper dental midline from the facial midline, upper lip curvature, smile line, smile arch, smile width, and shape of the maxillary central incisors), correlating them with age and gender. *Materials and methods*: Caucasian individuals (N = 114) were grouped by gender (male and female) and age (group I—18 to 30 years old; group II—31 to 50 years old; and group III—over 50 years old). Using a digital camera, extra and intraoral pictures were taken to analyze the variables above-mentioned. The data were statistically evaluated, considering a significance level of *p* < 0.05. *Results*: Most participants found deviations of the upper dental midline, straight upper lip curvature, and the medium smile line coincided with the facial midline. The parallel smile arch exposing 9 to 11 upper teeth, the absence of exposure of lower teeth when smiling, and oval upper incisors were prevalent parameters. Regarding gender, significant results were found for the curvature of the upper lip (*p* = 0.049), the smile arch (*p* = 0.001), and the shape of the upper central incisors (*p* = 0.004). For age, the association with the curvature of the upper lip (*p* = 0.032), the smile line (*p* = 0.001), the smile arch (*p* = 0.007), the width of the smile exposing lower teeth (*p* = 0.002), and the shape of the upper central incisors (0.012) were significant. *Conclusions*: Within this study’s limitations, gender and age affect the anterior teeth shape and upper lip curves; gender and age did not influence the coincidence between dental and facial midlines.

## 1. Introduction

Dental esthetics involves subjective point-of-analysis, which raises questions and debates for individual assessment [1]. On the other hand, esthetics must be guided by logical, scientific, and intuitive principles and techniques to create a pleasant smile [2,3]. Mouth (smile) and eyes are the most important social components involved with expression and facial appearance, [4] leading to an increasing demand for satisfied smiles [5]. Principles standardized for the facial esthetic involve alignment, symmetry, and correct facial (vertical) proportions [6]. A perfect integration between facial and dental components is required to find harmony in the smile, [7] which helps to establish and predict the esthetic result before the intervention, which encompasses soft and hard tissues of the face.

Also, professionals must consider other variables, such as age, gender, race, and social and cultural influence, which are decisive in creating smiles. Despite being partially guided by genetics, facial proportions are affected by aging, which can lead to an uneven facial esthetic [8]. Sarver and Ackerman [9] reported that a correct smile analysis should be based not only on three dimensions (frontal, oblique, and sagittal), but on four dimensions, also known as the time of life. This last dimension influences the ability to lift the upper lip, causing a reduction of the smile and leading to a consecutive decrease in exposure of the maxillary incisors [10,11,12]. In addition, the length of the upper lip can influence the amount of incisor exposure, decreasing the maxillary incisors’ exposition and increasing the mandibular incisors’ exposure [10,12,13]. A reduction in gingival exposure accompanies a decrease in maxillary incisor exposition. Then, knowing the dentofacial changes caused by aging is essential to obtain a better clinical result according to different age groups [14].

Another critical factor is gender. Women are characterized by smiling more expansively, with greater frequency and intensity than men [13]. In addition to the difference in the level of exposition of the incisors among different ages, men and women also have different patterns for the lip-incisal relationship [14]. According to the literature, when smiling, a standard exposure of the upper incisors in men is between 30% and 70%, whereas 70% to 100% is the interval found for women. More extensive gingival exposures are more acceptable in women, characterized by high and medium smile lines; on the other hand, men have low smile lines [15].

The perfect integration between facial and dental components is essential to achieve harmony in the smile. Thus, the aim of this investigation was to characterize a specific population regarding esthetic parameters of the smile, observing predominant patterns for each variable analyzed, and additionally, to correlate the parameters studied with the patients’ ages and genders. These standards can help clinicians in their work, pursuing a more natural and biomimetic rehabilitation.

## 2. Materials and Methods

### 2.1. Study Design and Selection Criteria

This observational study followed the Declaration of Helsinki (1975, updated 2013). After internal ethical approval (Universidade Católica Portuguesa, Viseu, Portugal), the patients from the university received an explanation about the study; they were evaluated and accepted the Informed Consent, permitting us to enroll 114 Caucasian participants. The participants were grouped by gender (male [M] and female [F]) and age (group I—18 to 30 years old; group II—31 to 50 years old; and group III—over 50 years old) to appraisal and classification regarding the variables studied.

### 2.2. Criteria of Eligibility

The inclusion criteria were: (i) healthy teeth without anomalies in shape, number, and size, (ii) teeth in the correct position, (iii) patient without previous orthodontic treatment, orthognathic surgery, or lip surgery, and (iv) age ≥ 18 years old. The following exclusion criteria were considered: (i) age under 18 years old, (ii) any anterior teeth extracted, (iii) restorations on the buccal face of anterior teeth, (iv) any prosthetic treatment in the anterior teeth and upper premolars, (iv) previous or ongoing orthodontic treatment, (v) presence of deciduous tooth, (vi) presence of periodontal disease or gingival hyperplasia, (vii) vertical maxillary excess or passive eruption, and (viii) the presence of bruxism or parafunction.

### 2.3. Digital Equipment and Standardizations

The participants were photographed by one author (JC), who used a background template (Supplementary Figure S1) for guidance and reference of the anatomical parameters (trichion, glabella, mentonian [horizontal], and bipupillary lines [vertical]). A Canon EOS 1200D^®^ digital camera was used with a 105 mm Macro lens and Yongnuo YN-14EX flash. The operator kept the same reference in the ground to take the pictures (1.5 m away from the wall for extraoral photos and 30 cm away for the intraoral photo). All of the photographs were taken in the same place. For intraoral photographs, manual mode was selected (f = 29; ISO = 200; shutter speed = 1/125), and for extraoral pictures, TV mode (ISO = 200; shutter speed = 1/125) was applied. Instructions were given to the participants to take two extraoral frontal photographs: (i) to have the lips relaxed and (ii) afterward, smiling. Intraoral photography was performed using similar retractors and conditions. The picture collection resulted in three pictures per participant (two frontal extraoral photos [resting and smiling] and one frontal intraoral), which were analyzed by another professional (PCL).

### 2.4. Variables Analyzed

All the following variables were assessed: (i) deviation of the upper dental midline from the facial midline, (ii) upper lip curvature, (iii) smile line, (iv) smile arch, (v) smile width–number of teeth exposure, (vi) shape of the maxillary central incisors, and (vii) lower teeth exposure. The parameters are better described in Table 1.

## 3. Statistical Analysis

The data obtained were statistically analyzed using the Statistical Package for the Social Sciences program (SPSS^®^, version 24). The chi-square test of independence was used to make the association between two variables, and Pearson’s chi-square test for three or more variables. The significance level considered was *p* ≤ 0.05.

## 4. Results

### 4.1. Sample Characterization

In total, 342 digital photographs analyzed, which were taken from 114 participants (68 females [59.6%] and 46 males [40.4%]) aged between 18 and 80. Of these, 40 subjects were included in group I (between 18 and 30 years old), 46 subjects aged between 31 and 50 years old were in group II, and 28 subjects formed group III (>50 years old).

### 4.2. Variables Analyzed

**Figure 1:**
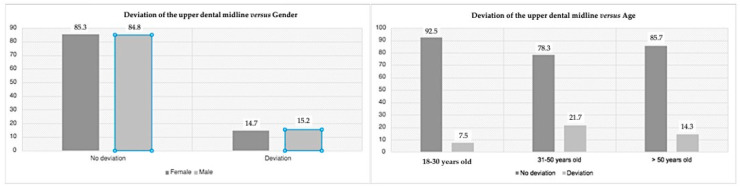
Prevalence of the deviation between upper dental midline and facial midline, according to gender (**left**) and age (**right**).

For all variables studied, Table 2 summarizes all main values and statistical results. The upper dental midline deviation compared to the facial midline did not relate to gender (*p* = 0.940) or age (*p* = 0.180). For this feature (Figure 1), most participants (97 individuals, 85.1%) did not have midline deviation; in 40 individuals aged between 18 and 30, 37 individuals (92.5%) did not present dental midline deviation; among the 46 individuals aged between 31 and 50, 10 individuals (21.7%) had a deviation; and among the 28 individuals aged over 50 years, 24 individuals (85.7%) did not present maxillary midline deviation. Gender (*p* = 0.940) and age (*p* = 0.180) were not related to the deviation of the upper dental midline.

**Figure 2:**
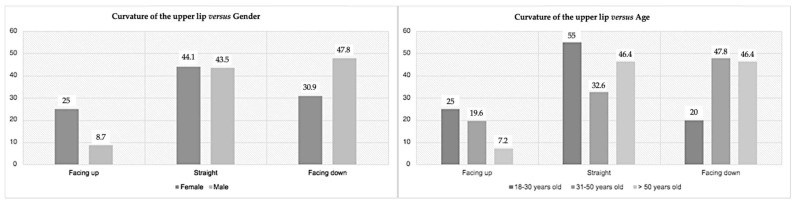
Prevalence of the upper lip curvature according to gender (**left**) and age (**right**).

On the other hand, the second variable studied (upper lip curvature) had significant results for gender and age (respectively, *p* = 0.049 and *p* = 0.032) (Figure 2). Of the 114 participants, 21 individuals (18.4%) had an upward curve of the upper lip, 50 individuals (43.9%) had a straight upper lip, and 43 individuals (37.7%) presented downward curvature of the upper lip. In females, the straight upper lip was the most prevalent (44.1%), while in males, the upper lip downwards was most prevalent (47.8%). A straight upper lip was the most prevalent in the younger class and the downward curve was the least prevalent. For elders, there was a greater prevalence of straight lips (women) and downward-facing curvature (men), with upward-facing curvature being the least prevalent.

**Figure 3:**
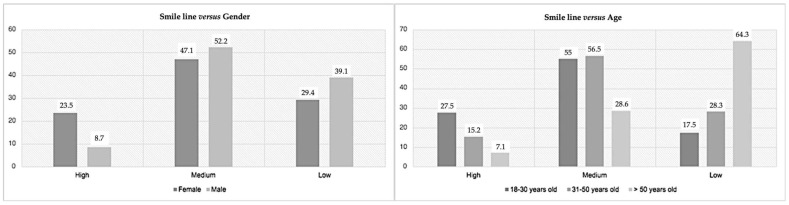
Prevalence of the smile line according to gender (**left**) and age (**right**).

When directly analyzing the smile line, 20 (17.5%) had a high smile line, 56 (49.1%) had a medium smile line, and 38 participants (33.3%) had a low smile line, which did not have a direct association with gender (*p* = 0.113). However, it was found to be a significant result when contrasted with age (*p* = 0.001) (Figure 3). Thus, the medium smile line was the most prevalent (around 56%) for younger individuals (up to 50 years old), but for those older than 50 years, the low smile line was the most prevalent (64.3%).

**Figure 4:**
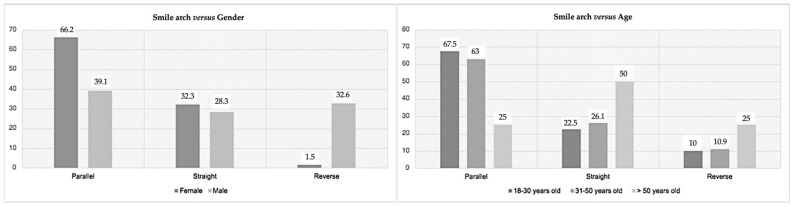
Prevalence of the smile arch according to gender (**left**) and age (**right**).

**Figure 5:**
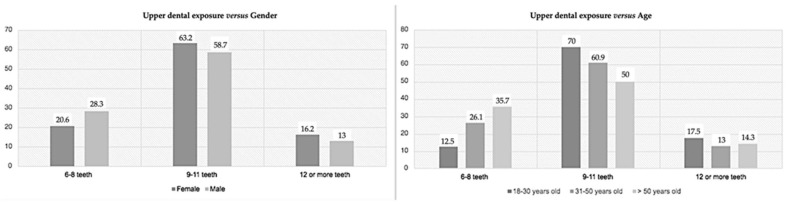
Prevalence of the upper teeth exposure according to gender (**left**) and age (**right**).

The smile arch and width were the other two parameters associated directly with the smile. Therefore, only the smile arch had significant statistical results for gender (*p* = 0.001) and age (*p* = 0.007) (Figure 4 and Figure 5). The parallel smile arch was the most prevalent (63 patients, 55.3%) in contrast to the reverse arch, which was the least prevalent (14%). In 45 women (66.2%), a parallel smile arch was found, and only one woman (1.5%) had a reverse smile arch. Oppositely, 39.1% of men had a parallel smile arch, and 32.6% had a reverse smile arch. In addition, the prevalence of the type of arch changes with age, with a parallel smile arch present in around 65% of the participants up to 50 years old, while 50% of the over 50-year-olds had a straight arch. Up to 50 years old, parallel smile arch was the most observed (more than 60%) and reverse smile arch the least (around 10%). Only for the sample with more than 50 years old, there was prevalence of straight smile arch (50.0%).

**Figure 6:**
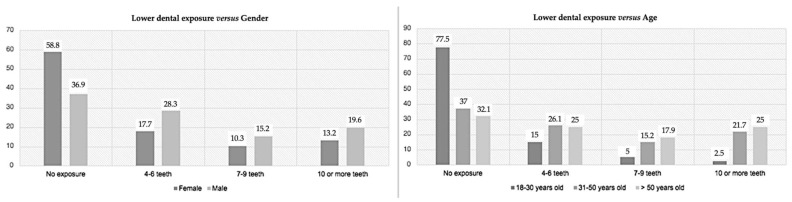
Prevalence of the lower teeth exposure according to gender (**left**) and age (**right**).

As mentioned above, smiling with upper teeth exposure (Figure 5) did not have statistically significant results either for gender or age (respectively, *p* = 0.620 and *p* = 0.257). In both genders, between 9 to 11 teeth exposed was more prevalent, which was similar for all the age groups; 12 or more teeth exposed was the least observed. On the other hand, when analyzing the lower teeth exposure (Figure 6), a significant correlation was found with aging (*p* = 0.002) but not with gender (*p* = 0.153). Older patients were significantly associated with the exposition of the lower teeth. In all groups evaluated, the non-exposure of the lower teeth was the most prevalent, 57 (50.0%) of the sample did not exposure the lower teeth, 21.9% exposed to 4 to 6 teeth, 12.3% exposed between 7 to 9 teeth, and 15.8% had exposure of 10 or more teeth. When comparing this variable with age, 31 younger individuals (77.5%) did not expose their lower teeth when smiling; for the group between 31 and 50 years old, 37.0% did not expose their lower teeth, while 12 individuals (26.1%) exposed between 4 and 6 teeth and 21.7% exposed 10 or more lower teeth when smiling; over 50 years, 32.1% did not expose their lower teeth, 25.0% exposed between 4 and 6 teeth, and 25.0% exposed 10 or more lower teeth when smiling.

**Figure 7:**
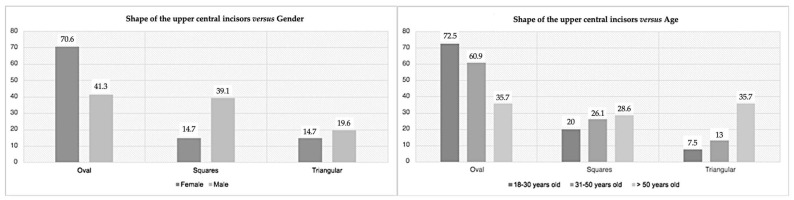
Prevalence of the shape of the upper central incisors according to gender (**left**) and age (**right**).

The last variable studied was the shape of the maxillary central incisors (Figure 7). It had significant results for gender (*p* = 0.004) and age (*p* = 0.012). Sixty-seven individuals (58.8%) were oval-shaped, 28 (24.5%) square-shaped, and 19 participants (16.7%) were triangular-shaped. According to gender, the most prevalent was the oval-shaped maxillary central incisors for women (70.6%), while for men, oval-shaped (41.3%) and square-shaped (39.1%) were similarly prevalent. Moreover, for the two younger groups, oval-shaped teeth prevailed, with the triangular shape being the least common; however, for over 50, oval and triangular-shaped teeth appeared to be the most prevalent, and square teeth represented the least prevalent shape.

## 5. Discussion

The advances in digital analysis, supported by several systems and software, seek to provide tools to guide and facilitate smile reconstruction. Nevertheless, it is imperative to understand the differences that exist between genders and the changes that occur due to age, as well as integrating individual patients’ characteristics, to obtain a harmonious result. Similar to the present study, several authors resorted to photographic records to analyze the smile, [5,12,16,17] characterizing the esthetic of the smile according to gender [16,17]. In addition, some age-related factors have also been discussed [18,19,20]. One of the most prevalent parameters was the assessment of the maxillary incisors exposition when resting and smiling, to verify the dentolabial relationship with aging. Thus, to fulfill the main objectives of the present study, the prevalence of different esthetic parameters of smiles was evaluated to assess whether they were significantly related to gender and/or age.

The alignment and symmetry between facial components, dental midline, and face proportions, is essential for a better esthetic result [21]. In agreement with the literature, our results found that the majority (85.1%) did not present deviation from symmetry [22,23]. Some reports mentioned that the upper dental and facial midlines coincided in 70% of the population, whereas the upper and lower dental midline did not coincide in almost three quarters [24]. Therefore, the prevalence of the coincidence reported between the maxillary and facial midlines was found in both genders, corroborating the results of other studies [5,16]. For all age groups, there was no midline deviation. Otherwise, the literature shows changes in dental position due to aging. However, these are not easily identified and become inconclusive due to the lack of long-term screening studies [25].

Lips are considered the smile control factor. Their evaluation is paramount when analyzing esthetics. According to our results, most individuals (43.9%) had a straight upper lip. Liang et al. [26] found a prevalence of the straight line in 39.9% of the cases, and Nold et al. [16] had a prevalence in 34% of the individuals. Dong et al. [27] reported that upward lip curvatures are rare, while downward (43%) and straight line (45%) curvatures proved prevalent. In women, a straight lip prevails, while downward curves characterize men. For younger people, the straight lip and the upward curvature were more encountered; however, with aging, there was a trend for the opposite (downward curve). Several factors can contribute to these characteristics, such as decreased skin elasticity and thickness, muscle fibers, and bone atrophy [28]. We assume that the loss of the vertical dimension of occlusion can also influence this factor because it can lead to loss of muscle tone and consequent skin sagging [29]. The upward and straight-line curvatures are considered more esthetics [30].

The smile line is an essential factor in dental esthetics because the higher it is, the greater the teeth and gum exposition [4]. The medium smile line was the most prevalent (49.1%). Our results were similar to Tijan et al. [17], who found a higher prevalence of medium smiles (68.94%). Nold et al. [16] also reported that most individuals had a medium smile line (52%), with 38% of the patients having a high smile and 10% having a low line. There were divergent results between our study and Nold et al.’s [16] study, which may be a consequence of the ages of the individuals enrolled in the sample, since the authors only included people between 19 and 29 years old. The medium smile line in this article prevailed in both genders. In contrast, several authors assume that high smile lines are an esthetic problem in males, with low smile lines as more prevalent. Oppositely, females were characterized by a higher smile line [4,16,31,32]. Under 50 years old, the medium smile line was the most prevalent, but over 50, the most prevalent smile line was low. Thus, we found data with statistical relevance that associates the smile line with age. Aging causes loss of muscle tone and lip flaccidity, causing an increase in lips length and resulting in less dental exposure [12]. This decrease attests to the tendency for lower smile lines with aging.

Smile arch can be designated differently according to the area of the literature analyzed (prosthesis, orthodontics, or esthetic dentistry) [33]. Frush and Fisher [34] defined the smile arch as the harmony between the curve formed by the incisal edges of the anterior maxillary teeth and the curvature of the upper edge of the lower lip. In our sample, 55.3% had parallel smile arches, whereas the reverse arch was the least prevalent. Nold et al.’s [16] results align with those obtained in our study; the authors presented 63% for parallel smile arches. Tijan et al. [17] had the highest percentage of parallel smiles (84.8%), with 13.88% presenting straight arches and 1.32% with reverse arches. Desai et al.’s [35] study also reported the prevalence of parallel over reverse smile arches, 48.4%, and 3.6%, respectively. On the other hand, Soares et al. [5], despite finding a minority of reverse smile arches, did not find differences between parallel and straight smile arches. Rodrigues et al. [36] reported that the presence of straight and reversed arches led to decreased esthetic perception for patients or dentists, similar to Carlsson et al.’s [37] results. Dong et al. [27] also reported that reverse arches were the least esthetically pleasing. Our results indicated that the parallel smile arch was the most prevalent for both genders; the reverse arch was the least prevalent in females, and the straight arch was the least prevalent in males. Goldstein [38] reported that the smile in elders showed more incisal edges of the anterior-superior teeth in a straight line, contrasting with younger smiles where the curvature is more accentuated. The more arched the curvature, the more youthful the smile; when it begins to be substituted by a straight line, the more aged the smile looks [39].

The number of teeth exposed in a smile can vary. In our sample, 61.4% of individuals exhibited between 9 and 11 teeth, 23.7% between 6 and 8 teeth, and 23.7% showed 12 or more teeth when smiling. When evaluating the smile width, Tijan et al. [17] found that the six anterior teeth and first premolars were mainly exposed in the upper arch (48.6%), followed by the exposure of the six anterior teeth with the first and second premolars (40.65%). Otherwise, Soares et al. [5] did not find differences between smiles until the second premolars and up to the first molars, and the lowest prevalence concerns the exposure of only six teeth. Nold et al. [16] found that, in their sample, 45% of the individuals exposed their teeth to the second premolar, 24% to the first premolar, 31% to the first molar, and none up to the second molar. Regarding the number of mandibular teeth exposed during smiles, we found no reports in the literature requiring the analysis of this parameter in future studies.

Correia et al. [12] evaluated the intercommissural distance, proposing that changes in this variable could explain the exposure of lower teeth in elders, suggesting that changes in the muscle tone might interfere with this distance. Al-Habahbeh et al. [40] and Dindaroğlu et al. [20] reported an inversion in the exposition of teeth, with a decrease for the upper teeth and an increase in the lower teeth exposition with aging. This event can be caused by the loss of lip elasticity, lower migration of the surrounding soft tissues, and the wear of the anterior teeth causing their shortening [22,23].

According to the shapes of the upper incisor (oval, square, and triangular), the prevalent central incisor had 58.8% oval shape, followed by the square (24.5%) and triangular (16.7%). Paranhos et al.’s [41] date suggest a different sequence; their data had the highest prevalence of square (31.37%), then oval shape (24.06%), and finally triangular (11.57%). Nold et al. [16] had a prevalence of 63% for an oval shape, 26% for a square, and 10% for a triangular shape, corroborating our results. Frush and Fisher [34] argued that rounded contours at the incisal angles are more harmonious in females. In contrast, straighter angles are more associated with males, supporting our findings. In younger classes, the triangular shape appeared as the least common; above 50, the oval and triangular shapes were equally found. Although we are unaware of studies on the prevalence of different dental shapes according to age, we think this relationship may be associated with tooth wear that alters the dental structure. The young central incisors are characterized by well-defined incisal and contact points, often at the incisal and middle third levels [9]. Aged teeth showed wear and tear on hard tissues due to phenomena (erosion, abrasion, or parafunctional habits) [42]. Thus, the width/height ratio is commonly altered, and the teeth become shorter and less visible [42]. It is important to remember that tooth shortening can result in the loss of vertical dimension, contributing to an older and less aesthetic appearance [26].

### Limitations of the Study

This was a pilot study that analyzed 6 variables for facial and smiles esthetic, intending to provide a base for future studies. Then, a limited number of participants (N = 114) were enrolled, which was satisfactory for this primary research. Also, the study was only conducted within the Caucasian race; for future studies, we suggest including other races to enlarge and increase the understanding of this topic. Moreover, we believe that more clinical parameters might be investigated regarding the smiles, such as in the skin and perioral muscles between younger and elders, labial proportions, intercommissural distance, changes in the dental structures due to wear processes, and the light incidence and reflex in different racial groups. Furthermore, our study was restricted to the smile analysis; however, future studies can consider analyzing the facial esthetic correlating smile with eyes, nose, and cheeks.

## 6. Conclusions

Within this study’s limitations, gender and age affect the anterior teeth shape and upper lip curves; gender and age did not influence the coincidence between dental and facial midlines. Moreover, according to the aging, triangular teeth were more evident. Also, young people had upward labial curves present, and older patients had a predominance of downward curves with a lower smile line, exposing more lower teeth when smiling. Furthermore, regarding gender, no statistical evidence was found matching it with upper and lower teeth exposure. In addition, women had a prevalent presence of straight lips, whereas men had downward lip curvature; oval teeth were more common in both genders. More clinical studies must follow with a more significant number of individuals to fully corroborate these results.

## Figures and Tables

**Table 1:** Description of the esthetic parameters evaluated.

Variables	Description	Pictures (Examples)
Deviation of the upper dental midline from the facial midline	To analyze whether there is a coincidence between the upper dental midline and the facial midline in all frontal smile photographs, by tracing the facial midline through the union of anatomical points: glabella, sub nasal, and cutaneous pogonion. To analyze the coincidence of this with the upper dental midline	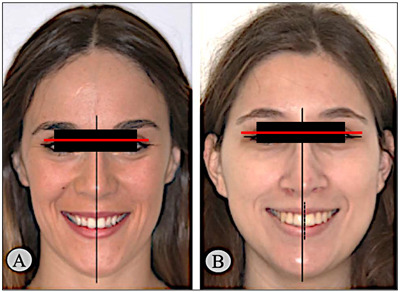 A—absence of upper dental midline deviation; B—presence of upper dental midline deviation.
Upper lip curvature	To analyze, in all smiles, if the curvature of the upper lip is facing up, if it is straight, or if it is facing down, by marking three points: one in the right commissure, one in the left commissure, and another in the central area of the lower edge of the upper lip.	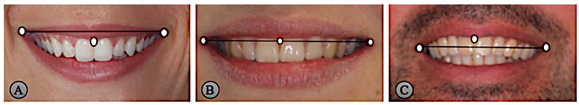 A—lip facing up; B—straight lip; C—lip facing down.
Smile line	To classify the smile line as high, medium, or low by evaluating the exposure of the anterior teeth during the smile. The high smile line is characterized by the exposure of the entire clinical crown of the maxillary anterior teeth together with a gingival band with approximately 3 mm of gingival tissue. The medium smile line occurs when there is exposure of 75% to 100% of the clinical crown of the anterior maxillary teeth as well as the interproximal gingival papillae. The low smile line presents less than 75% of the clinical crown of the maxillary anterior teeth without gingival exposure.	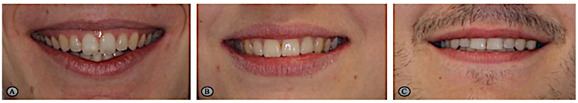 A—high smile line; B—medium smile line; C—low smile line.
Smile arch	To evaluate in all frontal photographs the existence of parallelism between the smile line and the upper edge of the lower lip by tracing of two curves, one formed from the union of the incisal edges of the anterior-maxillary teeth, and the other formed by the upper edge of the lower lip. After the tracing has been carried out, the classification of the relationship between the curves as parallel, straight, or reverse can be made.	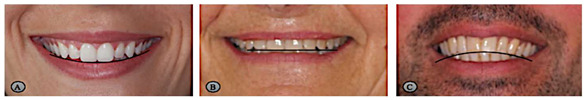 A—consonant smile arch; B—straight smile arch; C—reverse smile arch.
Smile width–upper and lower teeth exposure	The smile width corresponds to the number of teeth that are exposed in a smile, and is achieved by counting the number of teeth that each participant has exposed through the frontal photographs in smile.	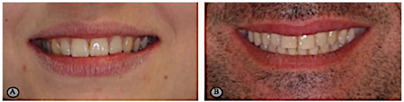 A—exposure of upper teeth; B—exposure of upper and lower teeth.
Shape of the maxillary central incisors	To classify the maxillary central incisors by observing the intraoral photographs according to their shape. Thus, these can be square (straight and parallel external limits forming a larger cervical area than the other shapes, with the incisal edge also being large), ovoid (more curved and rounded limits either incisal or cervical, accompanied by a decrease gradual from the incisal edge and the cervical zone), or triangular (lateral limits of the labial surface diverge towards the incisal, with the cervical area being narrower).	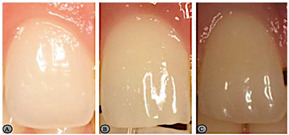 A—ovoid; B—square; C—triangular.

**Table 2:** Predominant values found for the variables analyzed per group.

	Gender			Age			
Variables	M (n = 46)	F (n = 68)	*p*-Value	18–30 y (n = 40)	31–50 y (n = 46)	>50 y (n = 28)	*p*-Value
Deviation of the upper dental midline (with deviation)	15.2%	14.7%	**0.049**	7.5%	21.7%	14.3%	**0.032**
The curvature of the upper lip (most common)	47.8% downward curve	44.1% straight lip	0.113	55% straight-line	47.8% downward curve	46.4% for straight lip and downward curve	**0.001**
Smile line	52.2% medium	47.1% medium	**0.001**	55.0% medium	56.5% medium	64.3% low	**0.007**
Smile arch	39.1% parallel	66.2% parallel	0.620	67.5% parallel	63.0% parallel	50.0% straight	0.257
Smile width—upper teeth exposure	58.7% 9–11 teeth	63.2% 9–11 teeth	0.153	70.0% 9–11 teeth	60.9% 9–11 teeth	60.0% 9–11 teeth	**0.002**
Lower teeth exposure	36.9% did not expose	58.8% did not expose	**0.004**	77.5% did not expose	37.0% did not expose	32.1% did not expose	**0.012**
The shape of the upper central incisors	41.3% oval shaped 39.1% square shaped	70.6% oval shaped	**0.049**	72.5% oval shape	60.9% oval shape	35.7% oval shape 35.7% triangular shape	**0.032**

y = years. Bold *p*-value = significative result (*p* < 0.05).

## Supplementary Materials

The following supporting information can be downloaded at: https://www.mdpi.com/article/10.3390/medicina59030595/s1. Figure S1: Background template for guidance and reference of the anatomical parameters.
