# Supplementary material for: The Interference of Age and Gender on Smile Characterization Analyzed on Six Parameters: A Clinical-Photographic Pilot Study
Source: Medicina (Kaunas). 2023 Mar 17;59(3):595. doi: 10.3390/medicina59030595 (PMC10053550; doi:10.3390/medicina59030595)
Supplement: Supplementary file 1 [file medicina-59-00595-s001.zip › medicina-2280480-supplementary.pdf]

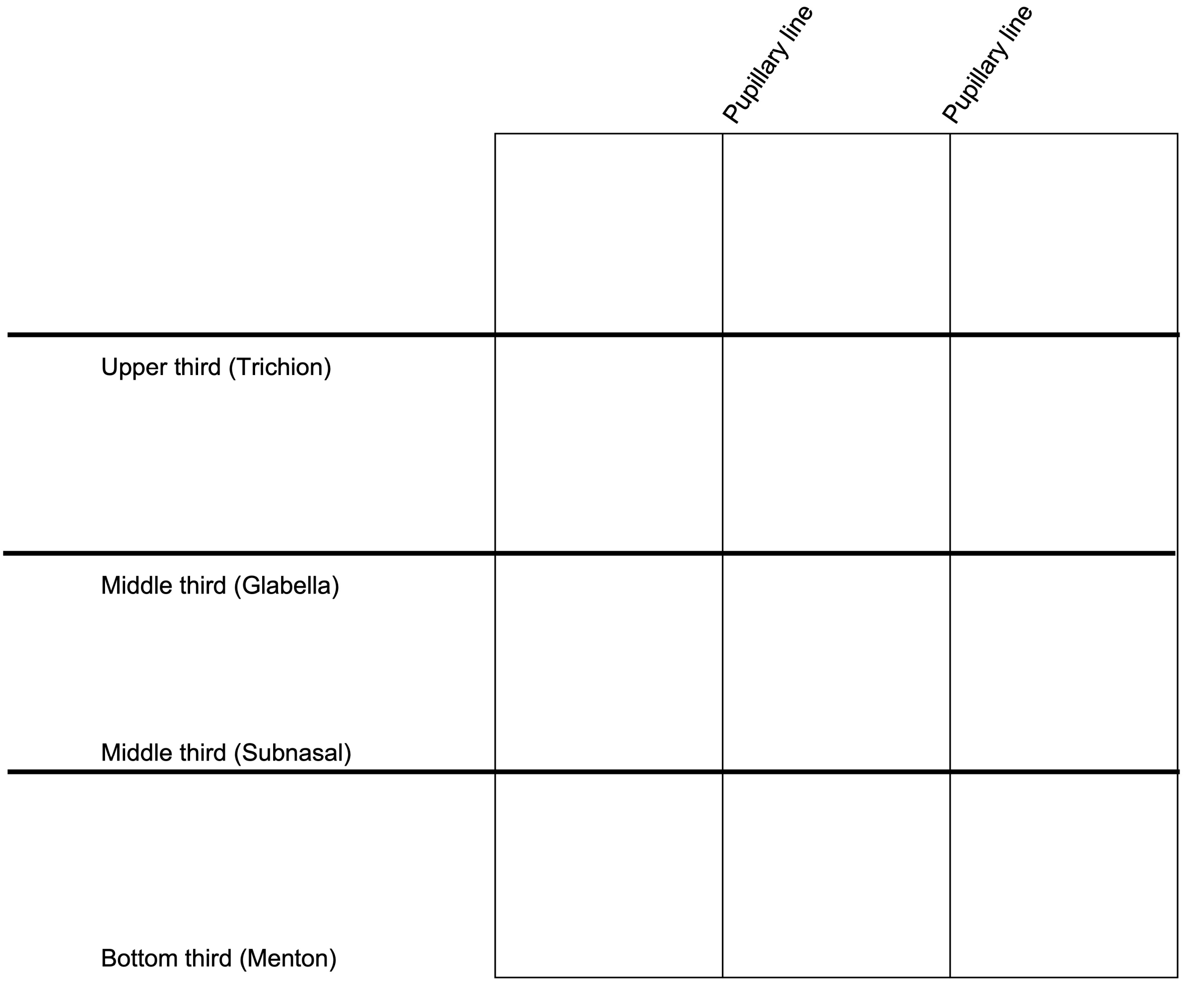

Figure S1. Background template for guidance and reference of the anatomical parameters.
